# Carcinosarcoma of the breast: two case reports and review of the literature

**DOI:** 10.1186/1757-1626-2-15

**Published:** 2009-01-06

**Authors:** Kristi M Esses, Ramona M Hagmaier, Susan A Blanchard, John J Lazarchick, Adam I Riker

**Affiliations:** 1 Department of Pathology, University of South Alabama Medical Center, Mobile, Alabama 36617-2293, USA; 2 Mitchell Cancer Institute-University of South Alabama, 1660 Springhill Avenue, Mobile, Alabama 36604, USA; 3 Diagnostic and Medical Center, 1700 Springhill Avenue, Alabama 36604, USA; 4 Gulf Regional Pathologists, Mobile Infirmary Medical Center, Mobile, Alabama, 36607, USA

## Abstract

Carcinosarcoma of the breast, often referred to as metaplastic carcinoma of the breast, is a rare malignancy with two distinct cell lines described as a breast carcinoma of ductal type with a sarcoma-like component. Clinically, carcinosarcoma of the breast is an aggressive breast cancer. The prognosis for carcinosarcoma of the breast is less favorable compared to more common types of breast cancer such as infiltrating ductal or lobular carcinoma. Currently, the evaluation of breast carcinoma includes hormone receptor analysis of the tumor tissue, with those positive for estrogen or progesterone responding better to both hormonal and chemotherapy.

Trastuzumab (Herceptin^®^) is available as an adjunct treatment for tumors which over-express the HER2/neu gene. Typically, metaplastic carcinomas of the breast do not express the estrogen or progesterone receptors and do not over-express the HER2/neu oncogene. As a result of this "triple negative" phenotype, such tumors tend to be more aggressive and are unlikely to respond to targeted therapy with Herceptin. The epidermal growth factor receptor HER-1/EGFR protein is expressed in the majority of metaplastic carcinomas and thus may serve as a potential therapeutic target for EGFR inhibitors such as gefitinib and cetuximab. The two cases we describe exemplify the aggressive nature of carcinosarcoma of the breast and support the findings that this tumor type does not express the common receptors found in other breast carcinomas. These case reports also emphasize the need for investigating the role for blockade of the HER-1/EGFR receptor with targeted therapies when found to be over-expressed in the primary tumor.

## Case presentation

### Patient 1

**Figure 1:**
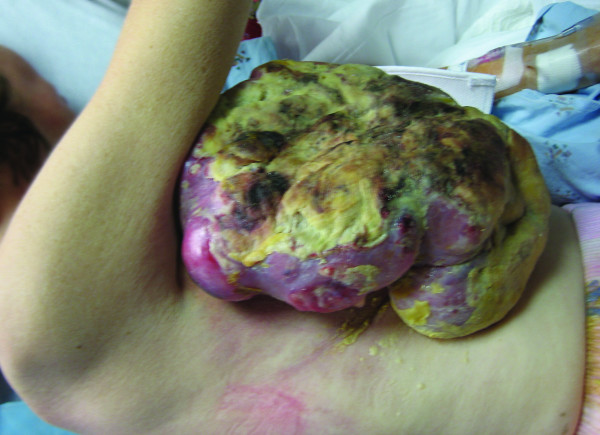
**Preoperative photograph of patient 1**.

This patient is a 48 year old female who was admitted with a large fungating right breast mass which measured 22 × 20 cm and elevated 12 cm above the skin surface (Figure 1). The patient stated that the mass began as a very small "bump" that she believed was a cyst. One year prior she had been evaluated by her primary care physician who described a bulging, red breast mass in the upper outer quadrant which measured at least 8 cm. Core biopsies were performed and the pathology revealed a high-grade malignant neoplasm with dominant features of carcinoma and a suggestion of carcinosarcoma. The patient chose to not undergo treatment and allowed the mass to enlarge with resultant ulceration of the mass through the skin with centralized necrosis and bleeding of the mass. The patient's associated symptoms included a 50 pound weight loss over the previous 6 months with intermittent low-grade fevers.

The patient's past medical history was significant for hypertension and left ear deafness. No significant surgical history was reported and her family history was not significant for malignancies in any first degree relatives. Physical examination revealed a cachetic female in significant pain with a large, extensively necrotic mass on the right anterolateral breast and chest wall. The necrotic tissue was on an erythematous, dense mound of tissue that extended to the lateral and central chest wall. No cervical, supraclavicular, left axillary or inguinal lymphadenopathy was appreciated on physical exam. There was palpable lymphadenopathy within the right axilla.

**Figure 2:**
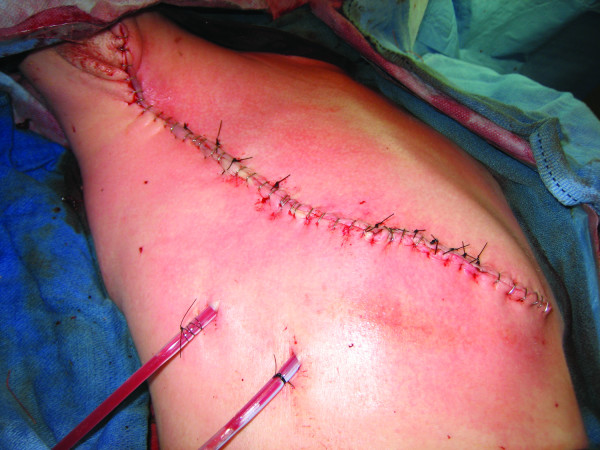
**Postoperative photograph of patient 1**.

The patient was taken to the operating room and underwent a right radical mastectomy. This encompassed removal of the entire pectoralis major and minor musculature down to the right chest wall, and a complete level III axillary lymph node dissection was performed. The skin flaps were viable, not grossly involved with tumor, and were able to be closed primarily (Figure 2). The patient recovered uneventfully and was discharged two days later. The patient developed a moderate amount of right upper extremity chronic lymphedema.

**Figure 3:**
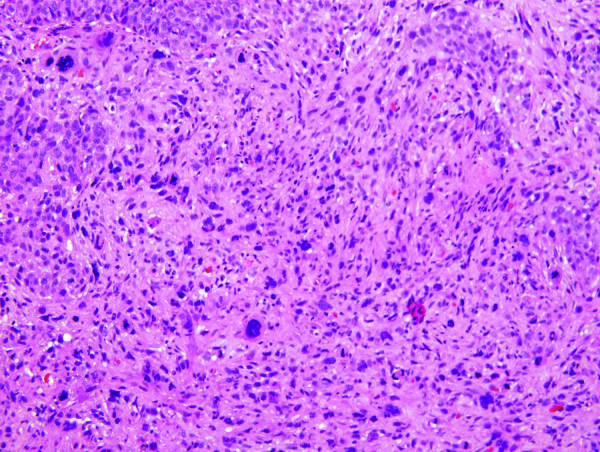
**Photomicrograph of carcinosarcoma (hematoxylin-eosin stain)**.

**Figure 4:**
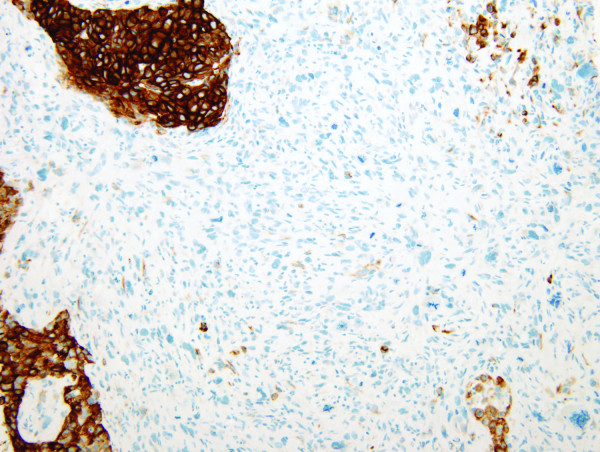
**Photomicrograph of carcinosarcoma (cytokeratin stain)**.

**Figure 5:**
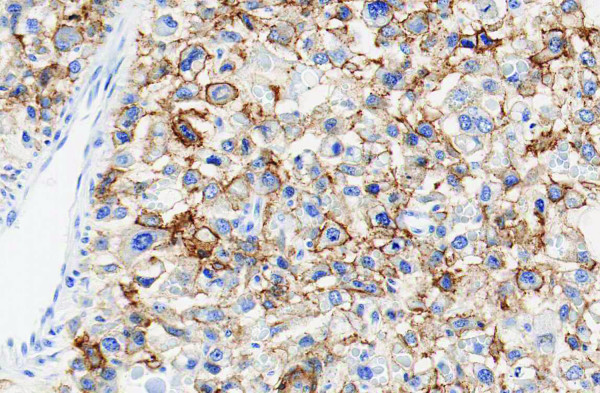
**Epidermal Growth Factor Receptor protein expression is analyzed by immunohistochemistry, staining positive**.

The final pathology of the right radical mastectomy revealed a high grade carcinosarcoma measuring 22 cm in greatest diameter (Figures 3 &4). There was extensive cutaneous ulceration with underlying dermal involvement by the tumor. The deep surgical margin of resection which included the pectoralis major and minor muscle was negative for tumor. A total of 2 of 28 axillary lymph nodes were positive for metastatic carcinosarcoma with extranodal tumor extension identified. All surgical margins were negative for tumor. Immunohistochemical profile revealed a neoplasm with a dimorphic histology. The epithelial (carcinomatous) component stained positive for cytokeratin immunostain CAM5.2, and the mesenchymal (sarcomatous) component was negative for CAM5.2, actin and desmin, but positive for vimentin. The tumor was estrogen and progesterone receptor negative and not amplified for the HER-2/neu gene via fluorescence *in situ *hybridization (FISH). Expression of HER-1/EGFR receptor was examined by immunohistochemistry with 70% of the cells expressing the EGFR protein, classified as moderate staining intensity (Figure 5). Based on available data and using AJCC criteria, the final pathologic staging was a T4b, N1a, Mx, Stage IIIb lesion.

A post-operative whole body PET/CT fusion scan and radionuclide bone scan showed no evidence of metastatic disease. As per NCCN guidelines regarding her stage of tumor, we recommended adjuvant radiation therapy to the chest wall and axilla in addition to chemotherapy. She received dose-dense doxorubicin and cyclophosphamide followed by paclitaxel and subsequently radiation therapy to the chest wall and axilla.

### Patient 2

This patient is a 51 year old female who was evaluated by her primary care provider for a tender left breast mass that had enlarged over the previous month. The patient's past medical history was significant for cervical cancer, and her family history was significant for her mother dying of ovarian cancer at the age of 57. The patient also reports two aunts with ovarian cancer who died at the ages of 69 and 59. On physical examination, a round, mobile mass measuring 2 cm was readily palpable at the 2 o'clock position of the left breast, 5 cm from the nipple areola complex. There was no palpable axillary lymphadenopathy. Subsequent mammography and ultrasound examination of the left breast revealed a hyperdense 1.6 × 1.5 × 1.1 cm mass within the 2 o'clock position, with ill-defined and angulated borders, assessed as a BIRADS category 4. There was a second lesion within the 11 o'clock position of the same breast, measuring 0.7 × 0.5 × 0.6 cm also considered suspicious for malignancy, assessed as a BIRADS category 4. An ultrasound-guided core biopsy was performed of the larger mass, revealing a carcinosarcoma with a few associated areas of fibroadenoma. Immunohistochemical staining revealed both an epithelial (carcinomatous) component which stained positive for cytokeratin CAM5.2 and a mesenchymal (sarcomatous) component that was negative for CAM5.2, but positive for vimentin.

The patient opted for a left simple mastectomy and right prophylactic simple mastectomy, with bilateral axillary sentinel lymph node mappings performed. The final pathology revealed the left simple mastectomy containing a 2.7 cm carcinosarcoma characterized by a grade 3 epithelial component with an associated, but separate, high grade mesenchymal component (Figures 3 &4). All sentinel lymph nodes and surgical margins were negative for malignancy. The final pathologic staging was a T2, N0, MX, Stage IIa. Hormone receptor assay revealed the tumor to be negative for both estrogen and progesterone receptors, with HER-2/neu also negative. Ki-67 expression was 60%, with expression of the HER-1/EGFR receptor with a 50% strong staining intensity (Figure 5). The patient recovered well and went on to receive adjuvant chemotherapy with dose-dense adriamycin (60 mg/m^2^) and cyclophosphamide (600 mg/m^2^), followed by a full course of Taxol.

## Discussion

Carcinosarcoma of the breast (metaplastic, biphasic metaplastic, metaplastic sarcomatoid carcinoma, sarcomatoid carcinoma) is an aggressive, rare neoplasm that has been reported to account for 0.08–0.2% of all breast malignancies [1-3]. Carcinosarcomas have been observed in various organs throughout the body, including the ovary and uterus. The true definition of metaplastic carcinoma of the breast is a tumor of malignant epithelial tissue (carcinoma) mixed with malignant cells of mesenchymal origin (sarcoma) with apparent histologic and cytologic features present on light microscopy and immunohistochemical testing [3-6]. Indeed, this tumor type is often referred to as metaplastic breast cancer, characterized as an unusual and uncommon neoplasm that is comprised by an admixture of two or more components [7]. The term carcinosarcoma was previously reserved for neoplasms where the demarcation between carcinomatous and sarcomatous components was distinct in all microscopic fields. The cells of origin for this neoplasm have yet to be agreed upon, but most research leads us to believe the cells are of myoepithelial origin. The tumor components may be homogeneously adenosquamous, or heterogeneously epithelial (adenocarcinoma) and mesenchymal (matrix, spindle cell and sarcomatous) in origin [7,8]. However, it seems more appropriate to term all breast carcinomas with obvious carcinomatous and sarcomatous features as biphasic metaplastic sarcomatoid carcinoma (MSC) [9].

Regardless of the name given to this entity, most metaplastic tumors of the breast are poorly differentiated, high grade, highly cellular, with mitotically active pleomorphic spindle cells. The majority are estrogen and progesterone receptor negative, and HER2-neu negative by immunohistochemistry [8]. The clinical and pathologic features of metaplastic breast carcinomas are important to distinguish from other types of uncommon breast malignancies such as spindle cell carcinoma, matrix producing carcinoma, fibrosarcoma, osteosarcoma, malignant fibrous histiocytoma, phylloides tumor and stromal sarcoma as their behavior, response to treatment and survival rates differ greatly.

Recently, Hennessy et al. reported on 100 patients with biphasic metaplastic sarcomatoid carcinoma (MSC) and 98 patients with carcinosarcoma identified through the SEER database. They compared clinical features and survival parameters for the two cancer types [9]. They conclude that both MSC and carcinosarcoma are aggressive, treatment-refractory tumors with shared clinical features and outcomes similar to poorly differentiated, receptor-negative adenocarcinoma of the breast. In comparing these two entities, they found that the initial T-stage of the tumor had a very strong association with overall outcome. They also identified significant differences in the metastatic spread capacity to regional nodal basins.

Clinical features of metaplastic breast cancer are similar to those patients with invasive ductal carcinoma. A recent review of 16 publications over a 21-year range (1984–2005) addressing the clinical and pathologic characteristics of MSC revealed a 5-year overall survival ranging from 49–68% [7]. However, when MSC was compared to a cohort of "typical" invasive breast cancer patients, the 5-year disease-free and overall survival was not significantly different (84% vs. 93%, 83%, 90%, respectively). Recurrence can be rapid as the primary tumor is aggressive, thus mandating close interval follow-up after resection. Pulmonary metastasis is more common than brain, skeletal or hepatic metastasis, and the prognosis for these patients is poor [10]. Outcomes for local recurrences are somewhat improved when surgical resection is achievable. In general, the recommended treatment options have followed the established NCCN guidelines for patients with invasive breast cancer. In the majority of the reported cases, mastectomy with or without axillary node dissection was performed, followed by post-operative chemotherapy and radiation therapy in various combinations.

Evaluation of patients with breast carcinomas includes analysis of the expression of various receptors on the primary tumor. The majority of carcinomas of the breast are estrogen receptor positive (estimated at 75%), progesterone receptor positive (estimated at 55%), and HER2 receptor is over-expressed (reported to be up to 25%) [7]. Adjuvant hormonal therapy and chemotherapy is based upon the receptor status of the primary tumor and is an important tool in treatment recommendations. The HER1/EGFR receptor is reported to be over-expressed in the majority of metaplastic sarcomatoid carcinomas of the breast and should be included in the initial evaluation of the various tumors which are described under this classification. The results may then be utilized to tailor the adjuvant therapy based upon these findings.

New treatment opportunities may exist with the development of agents targeting the EGFR receptor such as gefitinib (ZD1839, Iressa) and cetuximab (Erbitux). In a study of 20 cases of metaplastic carcinomas of the breast, they found that 14/20 MSC's were positive for EGFR expression, highlighting the potential utility of targeted therapies to the EGFR receptor [11]. Monoclonal antibodies and small molecule inhibitors of EGFR are currently being evaluated in clinical trials of patients with lung and colorectal cancer. It has been suggested that the frequent expression of EGFR in the absence of steroid receptors or other receptors of the EGFR family might render metaplastic breast carcinomas even more sensitive to EGFR tyrosine kinase inhibitors [12]. Further research needs to be performed in order to fully evaluate the potential of such therapy in patients with MSC.

## Conclusion

Obtaining an accurate diagnosis of MSC is essential in order to optimally tailor adjuvant therapy towards this aggressive breast cancer subtype. This requires an interdisciplinary approach to treatment, with the involvement of the entire oncology team comprised of the primary care physician, the pathologists, surgical oncologist, radiation oncologist, medical oncologist, and the supporting nursing and research staff. Adjuvant chemotherapy is generally recommended utilizing established NCCN guidelines for the more common types of breast cancer. By improving our understanding of MSC, we may provide such patients with novel and potentially effective treatment options that will ultimately translate into improved overall outcomes.

## Abbreviations

BIRADS: breast imaging reporting and data system; cm: centimeter; EGFR: epidermal growth factor receptor; FISH: Fluorescence in situ hybridization; IHC: immunohistochemistry; mg/m2: milligram per meter squared; MSC: metaplastic sarcomatoid carcinoma; NCCN: national comprehensive cancer network; PET/CT: positron emission tomography/computed tomography; SEER: surveillance epidemiology and end results

## Consent

Written informed consent was obtained from the patient for publication of this case report and accompanying images. A copy of the written consent is available for review by the Editor-in-Chief of this journal.

## Competing interests

The authors declare that they have no competing interests.

## Authors' contributions

KE was a major contributor in drafting the manuscript and provided the photographs. SB carried out the patient diagnosis. AR performed the surgeries and was a major contributor in drafting the manuscript. AR and RH were involved in the patient's post operative management. RH was a major contributor in writing the case report. JL conducted the histopathology. All of the authors were involved in the patient's care and all authors read and have been involved in approving the final manuscript.
